# Eye-tracking evidence of an association between social anxiety and avoidance of threatening faces in healthy women

**DOI:** 10.3389/fpsyt.2026.1712931

**Published:** 2026-03-05

**Authors:** Hanna Dietel, Taavi Wenk, Anette Kersting, Thomas Suslow, Vivien Günther

**Affiliations:** Department of Psychosomatic Medicine and Psychotherapy, Medical Faculty, Leipzig University, Leipzig, Germany

**Keywords:** attention, emotional facial expression, eye tracking, gaze, social anxiety

## Abstract

Socially anxious individuals are characterized by higher social fears and a sensitivity to signals of rejection. Eye-tracking studies in socially anxious participants without clinical diagnoses provided mixed findings on altered attentional processes. Investigations of gaze behavior in response to socially threatening scenes are scarce. By using eye-tracking technology in a free-viewing task, the present study examined in 108 healthy women the relationship between socially anxious tendencies and gaze orientation to anger and disgust faces and social scenes paired with neutral stimuli. The Liebowitz Social Anxiety Scale (LSAS) was used to assess social anxiety. Picture pairs were presented for 8000 ms to examine early (*probability of first fixation*) and late (*dwell time*) parameters of attention. To induce experiences of failure, participants were confronted with a social stressor. Social anxiety was not related to an initial attention orientation bias toward threatening stimuli, relative to neutral stimuli. However, dwell time bias for anger and disgust faces was negatively correlated with social anxiety. In non-patients, social fears and resulting avoiding behavior seem to go along with turning one’s attention away from negative facial expressions and more toward neutral stimuli. This attention pattern was not observed for socially threatening scenes. For faces, our results support the assumption of avoidance of threatening stimuli at later processing stages in high social anxiety. Alternatively, socially anxious individuals may show a relative attentional preference for neutral faces when they are shown simultaneously with threatening faces.

## 1 Introduction

Social anxiety is an intense, consistent fear of social situations, where the individual could be scrutinized by others. Socially anxious individuals are sensitive to negative judgements or signals of rejection by others, while they have a strong desire to make favorable impressions on people (1, 2). Socially anxious individuals can be characterized by a low self-esteem and a fear of embarrassing or humiliating themselves (3). Social anxiety can be seen as a personality trait, with individuals varying significantly in their tendency to experience it (2). Experiencing some discomfort and anxiety in social situations, such as during job interviews or public performances, is quite common and also occurs in the general population (2, 4). However, fear and experiences of distress in social situations, and resulting avoidance can become excessive and exert an adverse impact on daily functioning (5). According to Stein et al. (6) the lifetime prevalence for social anxiety disorder across all countries is 4%. With highest lifetime prevalence of 11-12%, social anxiety disorder is a relatively common disorder in Western high income countries (e.g., 7, 8).

Biological and environmental influences appear crucial in shaping social anxiety as a trait and a disorder (e.g., 9, 10). Genetic inheritance accounts for a proportion of individual differences in social anxiety, but there are also environmental factors that play a key role in its development and manifestation. Social experiences and circumstances, such as peer victimization, experienced exclusion, minority status, and parenting styles (11–14) have been identified as potential environmental influences that contribute to the expression of individual differences in social anxiety. There is also evidence that heritable factors (i.e., genetic risk variants) show complex interactions with environmental factors (i.e., negative parenting practices) to increase the risk for social anxiety (12). Cognitive models highlight attentional biases, meaning distinctive attention allocation toward threatening stimuli relative to neutral ones, as etiological factors of anxiety (15–17). It has been suggested that individuals with social anxiety have a higher tendency to focus their attention on potential social threats in their surroundings (16). This prioritized detection of negative signals is believed to occur early and automatically in the information processing stages. As Cisler and Koster (18) have summarized, most theoretical models predict a vigilance toward threat in anxious individuals. However, according to the authors, the reviewed models do not agree on components, underlying mechanisms and time course of attentional biases. Several theoretical models propose that anxious individuals not only exhibit an overactive threat detection system, but additionally demonstrate later attempts to regulate emotional distress through strategic avoidance of threatening information (18, 19). Avoidance is seen as a safety mechanism, helping individuals evade feared social situations and negative judgement (1). Armstrong and Olatunji (20) suggested that the initial second after a stimulus appears is primarily shaped by where attention is first directed and how gaze is initially maintained, restricting the opportunity to explore other regions of the stimulus. According to the authors, this early stage of attentional deployment is predominantly influenced by the stimulus’s external, exogenous features, while internally driven, endogenous control mechanisms emerge and gain influence as viewing continues. However, clear temporal definitions still need to be established in the eye-tracking literature regarding which specific parameters should be considered indicators of early versus late attention. According to meta-analyses based on eye- tracking studies (21), in clinical social anxiety disorder, there is weak evidence for early attentional biases toward negative facial expressions when they compete with neutral stimuli. A growing body of research illuminated altered attention as a function of socially anxious tendencies in individuals without a history of psychiatric disorders. However, eye-tracking studies using stimulus pairs are scarce and revealed heterogeneous findings. Evidence has been provided for late attentional biases toward negative facial expressions in individuals with high social anxiety (22, 23), but other studies failed to find altered attention patterns for threat as a function of socially anxious tendencies (24–27). Interestingly, the occurrence and direction of biases were shown to be influenced by the presence or absence of a situational stress condition. Garner et al. (25) have reported a faster orientation to emotional stimuli, followed by early avoidance tendencies in high social anxiety under the condition of an announced speech, indicating a vigilance-avoidance pattern. Singh et al. (28) have demonstrated early avoidance of anger faces in socially anxious individuals, regardless of the speech condition. However, results of this study did not indicate group differences between participants with high vs. low social anxious tendencies regarding attention allocation throughout the whole trial. In contrast, the induction of mortality thoughts, but not physical pain thoughts, appear to be accompanied by an early gaze bias toward threat faces in high social anxiety (29). In their qualitative review of eye-tracking investigations, Chen and Clarke (30) concluded that subclinical social anxiety can be characterized by an initial preference for, followed by avoidance of threat stimuli, when they are competing with neutral stimuli.

Notably, earlier studies that illuminated attentional processes during the simultaneous presentation of threatening versus neutral stimuli focused on facial expressions. Moreover, in the majority of experiments, anger faces were used as threat stimuli. The less frequent utilization of disgust faces as threatening stimuli is surprising, given that the expression of closed-mouth disgust signals interpersonal rejection and criticism (31) and that disgust is regarded as an affective component of hostility (32). Therefore, it has been argued that facial expressions of disgust may be highly salient for socially anxious individuals and may represent a perceived threat (33). To our knowledge, no previous study examined the influence of socially anxious tendencies on gaze behavior in response to scenes with a threatening social content compared to scenes depicting neutral social environments.

In the present study, we explored attention patterns to threatening facial expressions and scenes as a function of non-clinical social anxiety under the influence of a social stressor. To this aim, we administered a free-viewing task where threat stimuli were paired with neutral stimuli. Prior to the task, experiences of failure were induced by a difficult matrices test while the participant and the experimenter observed low performance and negative feedback. This experience of witnessed poor performance was expected to activate fears of failure and negative judgment.

Based on cognitive models of anxiety (18, 19), social anxiety was expected to be associated with an early gaze bias toward threatening faces and scenes (as indicated by a higher probability of first fixation). Moreover, it was hypothesized that highly socially anxious participants would allocate relatively less attention to threatening stimuli, as indicated by smaller dwell time biases.

## 2 Method

### 2.1 Participants

The final sample consisted of 104 women with a mean age of 22.94 years (*SD* = 3.35) and a mean number of 12.13 (*SD* = 0.42) school years. One hundred and eight women were initially enrolled for study participation, but three participants were excluded due to an unsuccessful experimental stress induction (see section Failure induction), and one participant was excluded because of familiarity with the experimental task. The participants were acquired using postings in university buildings and social media advertisement. The local ethics committee approved the study protocol. All participants gave their written informed consent and were financially compensated. There is evidence for sex differences in gaze patterns during the recognition of facial emotions (34). Moreover, women appear to demonstrate differential psychological and physiological responsiveness to social stressors (35). Therefore, only women were recruited for this study to collect a homogeneous sample.

### 2.2 Psychometric measures

With the Structured Clinical Interview for DSM-5 (36), past and current diagnoses of psychiatric disorders were ruled out in order to obtain a mentally healthy sample. Social anxiety during the past week was assessed using the German version of the self-report Liebowitz Social Anxiety Scale (LSAS; (37)). In this scale, 24 social situations are judged on a 4-point Likert scale based on the extent of anxiety (0 = none to 3 = strong) and avoidance behavior (0 = never to 3 = always) the described situation would provoke. The German version of the LSAS shows good to excellent internal and external validity, can be considered a standard instrument in the screening evaluation for SAD, and was widely used in previous SAD-related research (38, 39). In our sample (*M* = 34.75, *SD* = 15.88) the internal consistency was excellent (Cronbach’s *α* = 0.92).

The validated German version of the State-Trait Anxiety Inventory (STAI; (40)) was administered to assess current anxiety symptoms as a state. An item was added to the original questionnaire (“I feel like a failure”) to judge actual feelings of failure, resulting in 21 items for the scale. Internal consistencies before and after the stress induction were good (STAIpre: Cronbach’s *α* = 0.80; STAIpost: Cronbach’s *α* = 0.86).

### 2.3 Free-viewing task

**Figure 1:**
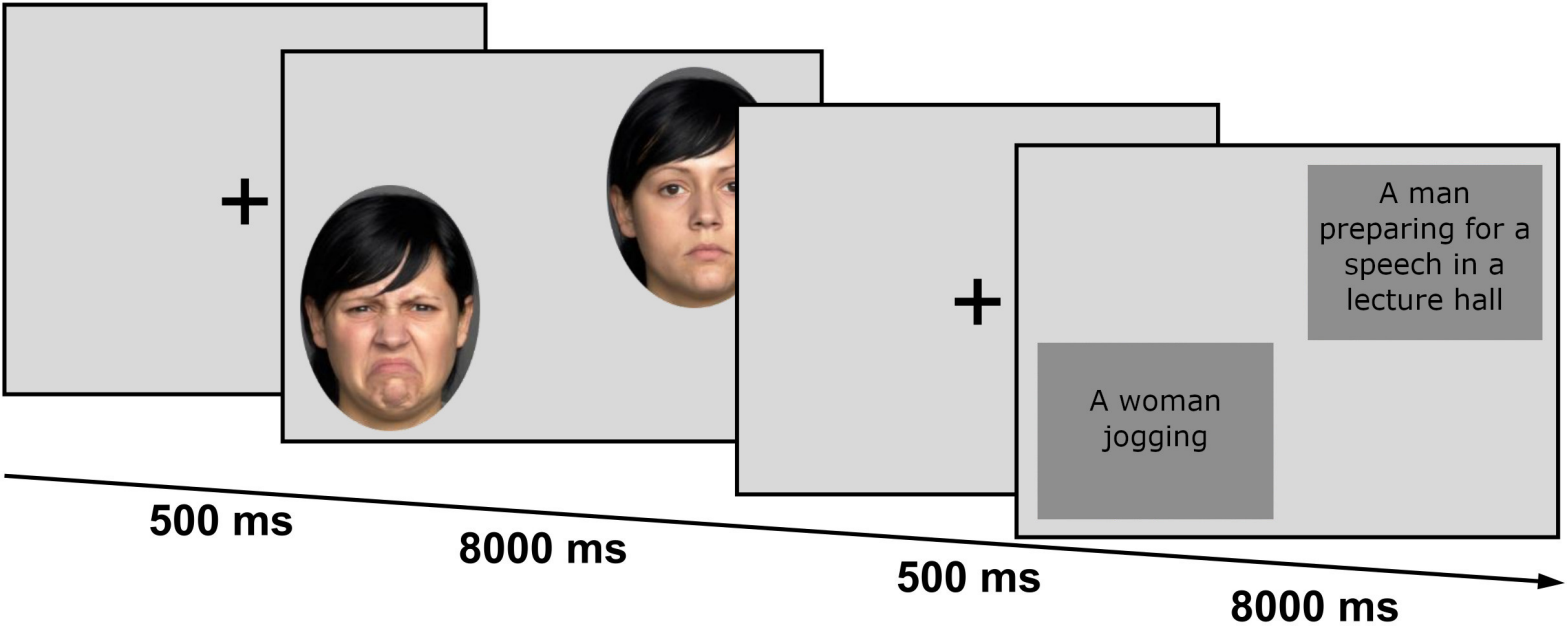
Free-viewing task. Depicted is the sequence of events within two trials of the eye-tracking experiment.

To assess participants’ attention allocation to emotional and neutral faces, colored photographs of 20 actors displaying neutral and (closed mouth) disgust expressions and of 20 different actors displaying neutral and angry expressions were obtained from the FACES database (41). Photographs were taken from white actors (20 men, 20 women) for each emotional expression under consistent lighting conditions. For each trial, a neutral face was paired with the disgust face (*n* = 20) or the anger face (*n* = 20) of the same actor. In some earlier studies, the neutral expression of an actor was shown more often than the emotional expressions throughout the experiment (e.g., 26, 27). To rule out the possibility that the occurrence of attentional preferences for emotional faces relative to neutral ones may, in part, be attributed to the fact that emotional faces represented novel stimuli, whereas the paired neutral face was already familiar, we presented both expressions of an actor equally often. To investigate participants’ attention patterns for threatening and neutral social scenes, 20 anxiety inducing (e.g., job interviews, giving speeches, anxious body symptoms) and 20 neutral images (e.g., street or indoor scenes with people) were selected from internet-based searches, the International Affective Picture System (IAPS; (42)), the validated Set of Fear Inducing Pictures (SFIP; (43)), and the Social Anxiety Picture Set - Muenster (SAPS-M; (44), partly based on the Emotional Picture Set (45)). Each fear-inducing scene picture was paired with a neutral scene picture, adjusted to their complexity and central objects. The threat pictures and neutral pictures were balanced with respect to their social content, i.e., both depicting people engaged in different activities. Pictures were taken from different stimulus sets to ensure an appropriate quality of the material and a familiarity with the ethnical background depicted in the photographs. The scene stimuli were matched by visual inspection with respect to their brightness and color. Pictures from internet searches were similar to pictures of the validated sets in content, but with a better resolution or higher cultural proximity^1^. Two psychologists chose the threatening and neutral scenes based on their potential to elicit fears in individuals with social anxiety. Sixteen healthy participants and thirteen patients with a diagnosis of social anxiety disorder (according to the SCID-5), who were all unrelated to the present study, evaluated the scene stimuli regarding their uncomfortableness, the induced discomposure, and anxiety. Ratings for neutral social scenes were significantly less negative and fear related (*M* = 4.93, *SD* = 0.80) than ratings for social threat scenes (*M* = 12.65, *SD* = 1.90; *t*(15) = -15.48, *p* <.001) in healthy participants and in patients (neutral scenes: *M* = 8.19, *SD* = 2.08; threatening scenes: *M* = 16.56, *SD* = 1.68; *t*(12) = -13.64, *p* <.001). All picture pairs were presented diagonally (i.e., the lower-left and the upper-right quadrant of the screen), with threatening faces or scenes appearing in each corner with equal frequency. All picture pairs were presented once against a grey background, with a random presentation order, resulting in a total of *N* = 60 trials. Participants were instructed via the computer screen to view the presented faces and scenes naturally, to allow the assessment of spontaneous attentional preferences. Each trial started with a fixation cross (black cross against a grey background), which had to be fixated for 500 ms. Subsequently, picture pairs were presented for 8000 ms, see **Figure 1**. Most previous studies presenting stimulus pairs used shorter presentation durations (< 3000 ms). We chose a longer presentation duration for two reasons. First, we wanted to ensure that late attentional processes could be genuinely assessed. Second, there is evidence that the psychometric properties of gaze behavior improve with longer stimulus presentation times [e.g., (27, 46, 47)].

### 2.4 Failure induction

Following the procedure of Tanzer et al. (48) we used experimentally induced failure as a social stressor. By administering a series of challenging matrices containing patterns of symbols, which required participants to identify the underlying rule to select the correct multiple-choice answer within a short period of time, participants were manipulated into thinking that they have failed an easily solvable intelligence test. The test was introduced as a really easy performance test, that all former participants aced quite successfully. Incorrect responses elicited failure feedback for the participant on the screen and were observed by the experimenter positioned close behind the participant.

The matrices test was administered before the eye-tracking task. To check for the success of the stressor, ratings on 5-point Likert scales were collected for subjective experiences of unpleasantness, insecurity, and stress immediately after the eye-tracking experiment. The participants were instructed to reflect on their feelings after the matrices test. Moreover, participants completed the STAI-state before the matrices test and after the eye-tracking experiment. Here, no instructions were given to report anxious feelings directly after the matrices test. Three participants were excluded from further analyses since STAI-state scores were not increased after the manipulation and participants stated that they were not unnerved or stressed at all. Of note, for seven participants, subjective ratings of stress were not available, but reports of increased state anxiety (STAI) indicated a successful manipulation of failure experiences for respective participants. Initial STAI scores in the sample (*M* = 34.58, *SD* = 5.62) significantly increased after the stressor and stimulus presentation (*M* = 40.28, *SD* = 7.46; *t*(103) = -9.44, *p* < 0.001). Of note, LSAS scores were not significantly associated with perceived stress (*r* = 0.06; *p* = 0.57).

### 2.5 Eye movement acquisition and eye-tracking parameters

The stimuli were presented on a 24-inch monitor (resolution: 1920 × 1200, refresh rate: 60 Hz). Gaze data was collected using a screen-based Tobii Pro Fusion eye-tracker at 120 Hz. A standard 9-point calibration procedure was conducted. Tobii Pro Lab software (version 1.232.52758, Tobii Technology, Stockholm, Sweden) was used for stimulus presentation and to record and analyze gaze data. Data from both eyes were used for analyses. For each trial, two areas of interest (AOI) were defined, one for the emotional picture and one for the paired neutral picture. One face stimulus subtended approximately 13.1° × 9.3° of visual (viewing) angle at a viewing distance of 70 cm and a scene stimulus subtended approximately 11.0° × 14.7°. The relevant field of the screen containing both stimuli subtended approximately 21.1° × 28.5° of visual angle for face pairs and 19.5° × 33.4°for scene pairs. No participant had to be excluded due to calibration issues.

The chosen eye-tracking parameter to investigate early attention allocation was *probability of first fixation* to the emotional picture, relative to the paired neutral picture. Thus, for each trial, the stimulus that received the first gaze fixation was determined for each emotion condition separately. The number of trials in which the threatening stimulus (e.g., disgust face) was fixated first was divided by the total number of trials for this emotion condition (*n* = 20), resulting in a proportion that reflects the likelihood of initial attention being directed toward the threatening stimulus. A probability of 0.5 indicates no preference for either stimulus (threat or neutral), whereas values above 0.5 indicate a bias toward the emotional stimulus. Visits are defined as the time between the start of the first fixation on the AOI until the end of the last fixation on the AOI, include fixations and saccades and, thus, correspond to the total amount of time spent looking within an AOI, also known as *dwell time*. Dwell time (total duration of visits) for the 8000 ms presentation period was extracted as an indicator of sustained or late attention. Dwell time represents the sum of durations from all visits on a specific AOI during a trial. Dwell time was computed separately for the emotional conditions for each AOI and each trial and then averaged for each participant. We computed attentional bias scores for dwell time by subtracting durations for neutral AOIs from durations for paired emotional AOIs. Higher scores indicate an attentional bias toward emotional stimuli. For interested readers, time to first fixation for emotional and neutral stimuli are provided in the **Supplementary Material**.

### 2.6 Data analyses

One-sample *t*-tests were computed for probability of first fixation and dwell time bias to analyze main effects of emotional relative to neutral pictures. Pearson product-moment correlations were conducted to examine associations between LSAS and attentional bias scores separately for angry and disgusted facial expressions and social scenes. Assumptions of the analyses were checked and found to be met [e.g., (49)]. Reported *p*-values are corrected for multiple comparisons with a false discovery rate (FDR) at 5% (50).

## 3 Results

### 3.1 Main effects

#### 3.1.1 Probability of first fixation

The probability of first fixation significantly differed from chance (50%) for disgust faces (*t*(103) = 2.59, *p*FDR = 0.03), but not for anger faces (*t*(103) = 0.83, *p*FDR = 0.48), or threatening scenes (*t*(103) = -0.72, *p*FDR = 0.48). The initial gaze was allocated to the disgust face more often than to the paired neutral face. Of note, despite a diagonal stimulus arrangement, 29% of participants demonstrated a strong preference for one side for their initial gaze, i.e., first gaze was directed to the emotional stimulus on one side with a probability higher than 80%, but with a probability lower than 20% if the emotional stimulus appeared on the opposite side. Excluding these participants from analyses did not alter the pattern of results.

#### 3.1.2 Dwell time bias

The bias in dwell time significantly differed from 0 for threatening scenes (*t*(103) = -3.76, *p*FDR < 0.001), but not for facial expressions of anger (*t*(103) = -1.55, *p*FDR = 0.12) or disgust (*t*(103) = -1.76, *p*FDR = 0.12). Across all participants, a negative bias score indicates that participants allocated relatively less attention to threatening scenes than to neutral scenes.

### 3.2 Correlations with social anxiety

#### 3.2.1 Probability of first fixation and social anxiety

LSAS scores did not significantly correlate with probability of first fixation to anger, disgust, or threatening scenes (see **Table 1**).

**Table 1:** Descriptive statistics [means (*SD* in brackets)] and correlations between eye-tracking parameters and social anxiety.

Eye-tracking parameters	*M* (*SD*)	LSAS
		correlation *r*
Early attention allocation		
Probability of first fixation		
Anger	0.51 (0.10)	-0.03
Disgust	0.52* (0.09)	-0.08
threatening scene	0.49 (0.08)	-0.04
Late attention allocation		
Bias dwell time (ms)		
Anger	-282.34 (1853.79)	-0.31**
Disgust	-362.50 (2095.59)	-0.33**
threatening scene	-405.01* (1099.35)	-0.16

*pFDR < 0.05, **pFDR < 0.01, asterisks in the *M* (*SD*) column refer to *t*-tests.

#### 3.2.2 Dwell time bias and social anxiety

**Figure 2:**
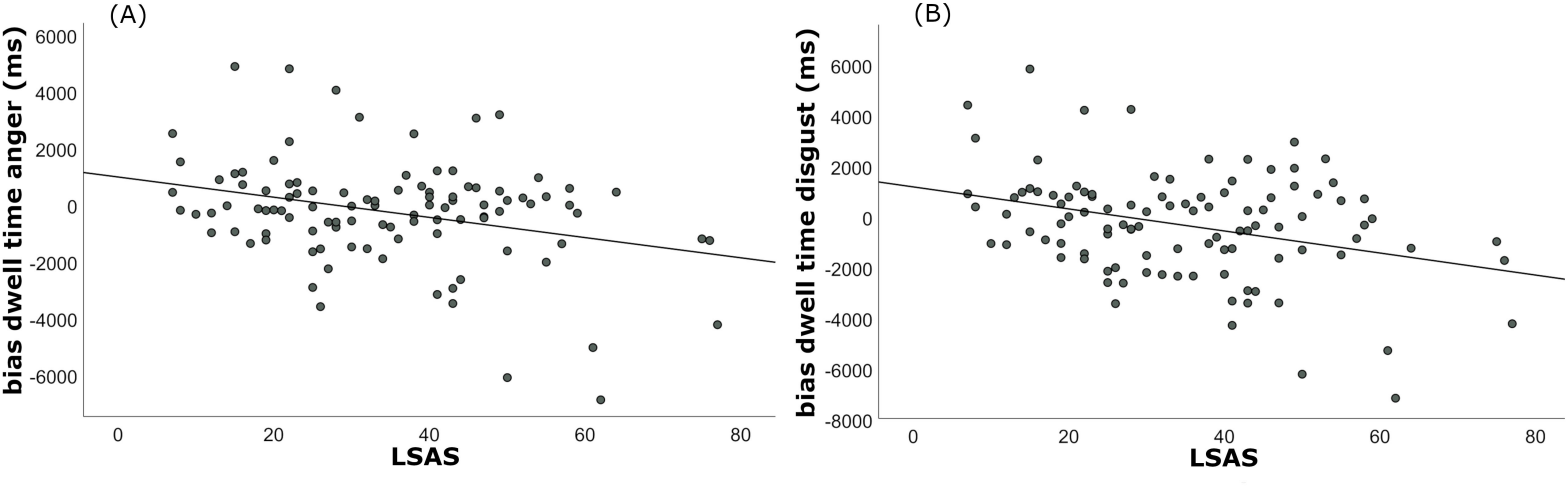
Relationship between social anxiety and dwell time bias. Social anxiety, as measured by the LSAS, was significantly negatively correlated with dwell time biases toward facial expressions of anger **(A)** and disgust **(B)**.

LSAS scores significantly and negatively correlated with dwell time bias for anger and disgust faces, but not for social scenes. Higher social anxiety was associated with shorter dwell times on threatening faces, relative to neutral faces, on later stages of attention allocation (see **Table 1**; **Figure 2**). To account for a potential modulatory effect of state anxiety on the correlation between LSAS and dwell time biases for threat, we calculated additional partial correlations with STAI state as control variable. The negative correlations for anger (*r* = -0.27, *p*FDR = 0.008) and disgust faces (*r* = -0.30, *p*FDR = 0.006) remained significant.

## 4 Discussion

Alterations in attentional preferences for potential threats in the environment are a key issue in popular cognitive theories of social anxiety (16, 19). Eye-tracking studies in healthy participants with varying socially anxious tendencies are scarce and provided heterogeneous findings. Apparently, the occurrence and direction of attention biases in social anxiety can be affected by experimentally induced stress (25, 29). The current eye-tracking study aimed to investigate early and late attention allocation to social-emotional stimuli in non-clinical social anxiety under the influence of a stressor.

In our study, there was an overall tendency for participants to direct their initial gaze on disgusted faces rather than neutral ones, but no significant main effects were revealed for initial gaze allocation to anger faces or threatening social scenes. Participants initially looked equally often at the angry faces and the threatening scenes as at the competing neutral stimuli. The latter findings contradict earlier reports in which healthy participants have shown an early preference for orienting their gaze toward happy and angry faces (23, 25). We can only speculate as to why an overall initial preference was found for disgust faces relative to neutral faces but not for angry faces. Disgust can signal social rejection, may be more ambiguous in its intention than anger, and may prompt rapid orienting in order to evaluate potential sources of exclusion and resolve uncertainty. In contrast, angry expressions signal direct interpersonal threat and are more confrontational than expressions of disgust. Accordingly, anger may initially attract attention in some participants but may also lead others to withdraw attention automatically in order to avoid provocation. Moreover, a significant dwell time bias indicated that participants looked on threatening social scenes for less time than to neutral social scenes. Those preferential tendencies were not significant for faces displaying anger or disgust.

Our data did not confirm the hypothesis that higher levels of social anxiety are characterized by an initial attention orientation bias toward threatening stimuli. Social anxiety was not correlated with a greater probability of first fixations on threat scenes or facial expressions of anger or disgust relative to neutral social stimuli. The absence of significant findings for the first gaze orientation is in line with Garner et al. (25) and Schofield et al. (23). Regarding the initial gaze preferences, Garner et al. (25) did not report group differences between individuals with high vs. low fear of negative evaluation in a dot probe task under the influence of a social stressor, although socially anxious individuals looked faster at angry faces when they were the target of the very first gaze. Furthermore, Garner et al. (25) demonstrated that individuals with more pronounced social anxiety had a lower probability to turn their first gaze at faces relative to the competing neutral objects, indicating an avoidance of faces. Our results could not confirm an early vigilance and increased perceptibility for threatening stimuli, which had been predicted in high social anxiety. However, our findings do not necessarily contradict cognitive models. Eye-tracking parameters for initial gaze orientation have been criticized for their low reliability, in part due to stable screen side preferences in first gaze direction (27). In addition, cognitive models of anxiety especially refer to clinical populations (15, 16), whereas our sample consisted of healthy individuals. Although there is preliminary evidence that non-clinical participants show similar attention patterns as patients with social anxiety disorder in a free-viewing task without a social stressor (51), our findings could not be generalized to clinical populations.

Our results support the hypothesis that highly socially anxious individuals look at threatening faces for relatively less time, as opposed to neutral faces. Partial correlations indicate that these relationships were not influenced by state anxiety. Individuals with more pronounced social anxiety may preferably turn their attention away from negative facial expressions of disgust and anger. Alternatively, social anxiety may be related to a greater allocation of attention to neutral faces when they are presented alongside threatening faces. Given that neutral facial expressions can be ambiguous (52) and may be interpreted as threatening by individuals with elevated social anxiety (53), highly socially anxious individuals may fixate longer on neutral faces in an effort to resolve this ambiguity. Our findings demonstrated that individuals with high social anxiety exhibited attentional biases for both angry and disgusted faces. Both emotions are considered affective components of hostility (32) and may represent highly salient and threatening stimuli for socially anxious individuals. In contrast, main effect analyses revealed that avoidance of socially threatening scenes, relative to neutral scenes, could be observed for all participants, regardless of their socially anxious tendencies. Our hypothesis that socially anxious tendencies are associated with relatively shorter dwell times on threatening scene stimuli was not supported. Relative to other stimuli, faces - along with their emotional expressions and focus of attention - are of central importance for humans and their non-verbal communication (54). Compared to scene pictures, the personal relevance of emotional faces with a direct gaze toward the observer might be particularly heightened in socially anxious individuals (e.g., 55). Through mutual gaze, the threatening message is conveyed directly to the observer and may trigger avoidance tendencies in individuals with high social anxiety. This is not the case for scene pictures, in which people were engaged in social interactions without a direct connection to the observer. Our findings contradict earlier eye-tracking studies with paired face stimuli, in which no effects of socially anxious tendencies on later attention parameters occurred (24, 26, 27) or biases toward angry or disgust faces were reported (22, 23). However, no stressor was implemented in these previous studies. Our results partly corroborate reports of Garner et al. (25), where highly socially anxious individuals under the influence of social stress showed diminished initial maintenance biases to angry faces relative to non-anxious individuals. Additionally, Singh et al. (28) observed an early avoidance of angry faces compared to neutral faces in individuals with strong fears of negative evaluation, irrespective of the presence or absence of a social stressor. Similar reduced early attention could be seen in high social anxiety when angry expressions were recognized in a facial crowd (56). Moreover, our findings of relatively reduced attention to threat are in line with a study using reaction time measures in a dot probe task and experimentally manipulated stress in non-clinical participants (57).

Heterogeneous findings in the literature might be attributed to several methodological differences. Most prior studies also recruited male participants, used shorter stimulus presentation durations, and did not experimentally induce social anxiety or failure experiences. Some studies on anxiety have reported opposing findings with regard to reduced versus prolonged maintenance of attention to emotional faces (58) or to the eye region of a confederate (59) when comparing men and women. Depending on the composition of a sample with regard to biological sex, effects reported in earlier studies may therefore be biased more toward decreased or toward increased attentional engagement. A potential moderator for the occurrence of vigilance and avoidance in social anxiety could be the absence or presence of a social stressor. The activation of an anxious mood state or fear of failure and negative judgement could be necessary before attentional avoidance of emotionally negative information can be observed. However, this explanation is speculative. Chen et al. (60) have demonstrated that socially anxious individuals avoid faces in an audience, but this effect only appeared when they were under the pressure of a speaking task. Our results are also congruent with findings from studies with clinical participants, in which socially stressed patients averted their attention away from (threatening) faces in an audience while giving a speech (61). In addition, socially anxious individuals were reported to avoid looking at the face of a conversational partner in a real-life social situation (62).

Clark and Wells (1) have argued that diverting attention away from social environmental cues prevents socially anxious individuals from disconfirming their false beliefs about an overly critical social environment and from getting an appropriate impression of the real danger of a social situation. The deployment of attention away from negative facial expressions could also reflect submissive behavior toward unfriendly persons, serving the purpose of avoiding a potential social confrontation or signaled rejection (63). Although several participants in our study reported slightly elevated levels of social anxiety and avoidance behaviors in social situations, they did not suffer from those anxiety symptoms and were not functionally impaired in their daily lives. Attentional avoidance strategies for negative information are considered a method to reduce state anxiety and emotional distress (18). Thus, one might speculate that attentional avoidance of unsettling social information, such as negative facial expressions, can be a coping mechanism to handle induced negative feelings of failure in healthy socially anxious individuals. Patients with social anxiety disorder have been successfully trained to allocate their attention away from negative faces, and these bias modification trainings could reduce anxiety symptoms (64, 65). Given this background information, spending relatively less time looking at angry and disgusted facial expressions does not necessarily have to be interpreted as a maladaptive coping mechanism in healthy socially anxious individuals. It may also indicate a successful strategy to deal with environmental social stress. The long-term social implications of the reported avoidance strategies remain unclear and require further investigation in longitudinal studies.

Our findings should be considered in the context of several additional limitations. Only healthy and young women were enrolled, so our results cannot be generalized to healthy male, older female, or psychiatric populations. When compiling the stimuli, we did not take into account that male and female faces might have different effects on attentional bias in female participants. There is evidence in women that attentional biases related to anxiety differ for female and male face stimuli, with anxious women showing relatively reduced attention only for same-sex stimuli (66). It is conceivable that bias effects are stronger, or even different, when only female or only male stimulus sets are used in future studies. Moreover, we did not recruit a control group that completed the free-viewing task without a social stressor. Thus, the impact of the presence or absence of a failure experience could not be investigated. To avoid arousing participants’ suspicion about the true purpose of the difficult matrices task, stress and state anxiety were assessed after the eye-tracking experiment rather than immediately after the test. Due to the retrospective perspective on perceived stress and the delayed measurement of state anxiety, slight biases in the assessment of the actual stress may have occurred. As the brightness of the images was not technically measured, image luminosity of the picture pairs was matched solely by visual inspection. Despite the implementation of a diagonal stimulus arrangement in line with the recommendations of Waechter et al. (27), a number of participants demonstrated a marked bias toward initiating gaze either on the lower-left or the upper-right region of the display, irrespective of the emotional content. These robust spatial biases in early visual orientation might have masked more nuanced effects of social anxiety on early gaze behavior. Consequently, the assessment of early attentional mechanisms remains difficult, as initial gaze metrics are characterized by limited reliability (27) that is not easily mitigated through current stimulus design strategies. Future studies may also clarify whether attentional avoidance tendencies in response to facial expressions of anger and disgust are observed in individuals with clinical social anxiety when they are confronted with a failure experience. In addition, it would be interesting to present other facial expressions that might be perceived as socially threatening, such as contempt. There is evidence that socially anxious individuals, under certain (stress) conditions, perceive positive facial expressions more negatively [e.g., (67)]. In addition, fear of positive evaluation is a prominent feature of high social anxiety (68). Therefore, the use of positive and affirming faces may be particularly informative when investigating attentional biases under the influence of a social stressor. To this day, no eye-tracking study has investigated attention toward pictures of anxiety-provoking (versus neutral) social scenes in patients with social anxiety disorder. Although we did not observe a significant correlation in healthy participants, future research may examine whether clinical social anxiety is associated with altered attention in response to socially threatening scenes.

In summary, we demonstrated in our study that in healthy individuals, social fears and tendencies to avoid these feared situations in everyday life go along with relatively reduced attentional engagement with facial expressions of anger and disgust. These information-processing biases occurred under the condition of social-evaluative stress, indicating that a threatening social context might be conductive to evoke altered attention patterns in high social anxiety.

## Data Availability

The datasets presented in this article are not readily available because participants did not provide consent for their data to be shared openly, and their consent was limited to scientific use only. Requests to access the datasets should be directed to the corresponding author.

## Footnotes

1 The study was conducted in Germany with predominantly Caucasian participants. Therefore, a picture in a conference room filled with an Asian audience was replaced with a picture showing mainly Caucasian observers.
