# Supplementary material for: Eye-tracking evidence of an association between social anxiety and avoidance of threatening faces in healthy women
Source: Front Psychiatry. 2026 Mar 5;17:1712931. doi: 10.3389/fpsyt.2026.1712931 (PMC12999869; doi:10.3389/fpsyt.2026.1712931)
Supplement: Supplementary file 1 [file DataSheet1.pdf]

Supplementary material

Table S1: Means and SDs for time (latency) to first fixation.

	AOI threat	AOI neutral
	M (*SD*) in ms	*M* (*SD*) in ms
Time to first fixation anger-neutral	230.22 (45.02)	225.19 (39.07)
Time to first fixation disgust-neutral	224.90 (41.42)	230.71 (53.15)
Time to first fixation threat-neutral scene	219.63 (48.98)	222.79 (40.83)

Of note, only data from trials in which the respective AOI was visited first were included. That is, if a participant's first gaze fell on the emotional picture, no time-to-first-visit parameter was calculated for the neutral stimulus on that trial, and vice versa.
